# Using Noise and Fluctuations for In Situ Measurements of Nitrogen Diffusion Depth

**DOI:** 10.3390/ma9100819

**Published:** 2016-10-05

**Authors:** Cornel Samoila, Doru Ursutiu, Walter-Harald Schleer, Vlad Jinga, Victor Nascov

**Affiliations:** 1 Department of Materials Science, Transylvania University of Brasov, Brasov 500036, Romania; 2 Department of Electronics and Computer Science, Transylvania University of Brasov, Brasov 500036, Romania; udoru@unitbv.ro; 3 Heat Treatment Department, SKF, Schweinfurt 97421, Germany; schleer@walter-harald.de; 4 Department of Electronics and Computer Science, Transylvania University of Brasov, Brasov 500036, Romania; jingavlad@gmail.com; 5 Department of Material Science, Transylvania University of Brasov, Brasov 500036, Romania; navirovi@gmail.com

**Keywords:** diffusion, furnace, magnetic, manufacturing, nitriding, sensors, temperature, thermo-chemistry

## Abstract

In manufacturing processes involving diffusion (of C, N, S, etc.), the evolution of the layer depth is of the utmost importance: the success of the entire process depends on this parameter. Currently, nitriding is typically either calibrated using a “post process” method or controlled via indirect measurements (H_2_, O_2_, H_2_O + CO_2_). In the absence of “in situ” monitoring, any variation in the process parameters (gas concentration, temperature, steel composition, distance between sensors and furnace chamber) can cause expensive process inefficiency or failure. Indirect measurements can prevent process failure, but uncertainties and complications may arise in the relationship between the measured parameters and the actual diffusion process. In this paper, a method based on noise and fluctuation measurements is proposed that offers direct control of the layer depth evolution because the parameters of interest are measured in direct contact with the nitrided steel (represented by the active electrode). The paper addresses two related sets of experiments. The first set of experiments consisted of laboratory tests on nitrided samples using Barkhausen noise and yielded a linear relationship between the frequency exponent in the Hooge equation and the nitriding time. For the second set, a specific sensor based on conductivity noise (at the nitriding temperature) was built for shop-floor experiments. Although two different types of noise were measured in these two sets of experiments, the use of the frequency exponent to monitor the process evolution remained valid.

## 1. Introduction

In the manufacturing of aircraft and automotive components, bearings, turbines, and other products, the nitriding process plays an important role [1,2] in ensuring dimensional stability. This is because there is no significant change in size at the structural level and only slight volumetric changes occur at the surface because of the diffusion of nitrogen [3,4].

The structure and chemical composition of nitrided surface layers is controlled by tuning the nitriding conditions, namely, the temperature and composition of the reactive gas. Currently, nitriding is typically controlled either using a “post process” method or via indirect measurements (H_2_, O_2_, H_2_O + CO_2_). The operating principle of a hydrogen probe is based on the thermal conductivity of H_2_. When oxygen is the monitored substance, its partial pressure is measured. Several types of sensors have been developed based on these two measurement principles. This paper presents a novel principle for monitoring the nitriding process using noise and fluctuations.

The physical nature of noise is related to physical values that define the macroscopic condition of materials, which are random variables because of the microscopic movements of atoms and molecules. The fluctuations of these values result in “parasitic” signals called *electronic noise*. The fluctuations of a single physical value give rise to a type of noise called *elementary noise* [5,6]. The primary physical parameters whose fluctuations generate elementary noise are electric charge, electric current, electric polarization, magnetization, and number of carriers.

Methods of studying noise originally emerged primarily as a practical necessity for achieving electronic circuit components that offer high performance in terms of their noise characteristics. The investigation techniques used to analyze these types of noise have since been extended from the study of fluctuations in electric circuits to the study of fluctuations in the physical parameters mentioned above. This extension is related to the fact that the fluctuations in the parameters that describe macroscopic physical systems may reveal various properties of those systems that cannot be derived by studying only the average parameter values. As a result, novel methods of researching the properties of certain materials and their fluctuations have emerged and have witnessed significant developments. Our team is recognized as the often-cited pioneers of the analysis of LED fluctuations [7].

The first researchers to suggest measuring noise to obtain information on materials after processing were Neri, Gotwald, and Szentpali [8]. Observations of conductivity noise in polymers have demonstrated that the noise spectrum is sensitive to the chemical environment [9]. A low-noise cantilever deflection sensor has been shown to increase the sensitivity limit of classical measurement techniques based on thermal Brownian motion. Molecular-resolution Atomic Force Microscope images of polydiacetylene single crystals showing insensitivity to the environment (air, vacuum or water) were reported. These authors obtained a more selective response from metallic oxide gas sensors using noise spectroscopy. The conductance of these sensors has a cut-off frequency that is four orders of magnitude lower than that achieved when mobility fluctuations are not considered. To increase chemical sensitivity, fluctuation-enhanced sensing (FES) was developed, in which the noise of a carbon-nanotube-based sensor is treated as a source of chemical information.

For the present study, from among the multitude of possible fluctuations (in balance, imbalance, ambient atmosphere, etc.), 1/*f* noise was chosen. Although a considerable number of tests and models have been described [10,11] to explain 1/*f* noise, none has received unanimous recognition. The spectrum of this type of noise was empirically described by Hooge as shown below:

(1) $$\frac{S_{I}\left(\omega \right)}{I^{\beta }}=\frac{S_{U}\left(\omega \right)}{U^{\beta }}=\frac{C_{1/f}}{f^{m}},$$

where *I* is the current (A), *U* is the voltage (V), *f* is the frequency (s^−1^), $S_{I}\left(\omega \right)$ is the power spectrum of the current fluctuations, $S_{U}\left(\omega \right)$ is the power spectrum of the voltage fluctuations, $C_{1/f}$ is the noise constant, and *m* is the frequency exponent. Here, $C_{1/f}=\alpha /N$, where α is a dimensionless constant (*α* ≈ 2 × 10^−3^), and *N* is the total number of charge carriers; however, this expression is questioned by researchers.

An apparently generally accepted notion concerning 1/*f* noise is that it represents fluctuations in conductivity. The nitriding sensor presented in the paper for real-time process monitoring is based on this property.

Regarding the theory of electronic noise caused by fluctuations, researchers generally accept the following expression [7,8]:

(2) $$S_{v}(f)\approx \frac{C_{1/f}}{f^{m}},$$

where $S_{v}(f)$ is the noise power spectrum density, and the exponent *m* is the slope of the regression line of the spectral analysis curve for the acquired signal. In our nitriding experiments, the goal was to determine this slope and to plot it against the nitriding time. As a result of these experiments, a linear relationship between the exponent *m* and the nitriding time was derived.

## 2. Materials and Methods

Steel samples (35CrNiM06) nitrided in a chamber furnace were used in laboratory investigations conducted to determine whether the frequency exponent *m* could be measured from the Barkhausen noise [12,13,14]. Barkhausen noise appears when samples are subjected to low-frequency variable magnetization in a hysteresis cycle (passing through saturation). Barkhausen noise is a non-stationary process. To obtain data on the magnetization dynamics, the excitation frequency *f* must be much lower than the lowest frequency measured in the noise spectrum. Under circumstances fulfilling this condition, the shape of the noise power spectrum turns out to be independent of the oscillation speed of the field. In this study, to ensure the accuracy and stability of the measurements, the noise signals were synchronized with the excitation signals (magnetization cycle) applied to the samples. The noise signals induced by the excitation signals were measured for 20 to 200 cycles. Error calculations performed based on these tests indicated that a measurement duration of 40 cycles was sufficient to ensure the accuracy and reproducibility of the measurements.

Because the nitriding process changes the composition of the surface layer of a treated sample, preliminary tests were run using Barkhausen noise. As shown above, the frequency exponent *m* and the noise constant α vary linearly, and this relationship may be used to monitor the diffusion process.

**Figure 1:**
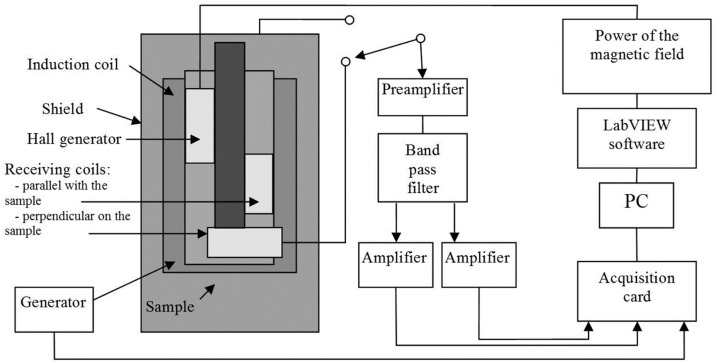
Laboratory setup for Barkhausen noise measurements [14].

Here, a comment on the morphological complexity of the nitrided layer is required. The Barkhausen signals from a material are known to depend on its crystalline microstructure. Steels subjected to nitriding contain pearlite and ferrite, and Barkhausen noise is sensitive to these constituents. For the ultimate goal of the laboratory tests—namely, finding a noise parameter that enables the monitoring of the diffusion of nitrogen—this sensitivity could be a disadvantage. Consequently, in the laboratory tests, efforts were made to ensure that all parameters that could influence the Barkhausen were kept constant: a single type of steel, a single type of heat treatment process, a single nitriding temperature, and a single diffusion length were chosen. The surface morphology was found to remain reasonably constant, showing a white porous layer and diffusion areas with segregated regions of iron nitrides. The existence of these regions indicated an increase in the volume of the diffusion layer with respect to the core, leading to the emergence of internal tensions, to which the Barkhausen noise is also sensitive. An attempt was made to select samples of similar morphology and similar tensions in the diffusion layer to ensure that only nitriding noise-affecting parameter would need to be considered. At no time was building a final sensor based on Barkhausen noise considered due to the sensitivity of this type of noise to layer morphology and tension. In addition, the magnetization processes that occur at higher temperatures were also considered an impediment because of the resultant changes in the magnetic characteristics of the steel. All of these considerations led to the decision to design a sensor based on conduction noise; in such a sensor, the electrode impedance will typically remain constant and will not change unless the composition of the electrode surface changes as a result of diffusion. The experimental setup shown in Figure 1 was used to measure the Barkhausen noise.

The system was composed of a “Unipan 237” nano-voltmeter calibrated for low-frequency noise, a sinusoidal field generator operating at a frequency of 0.2 Hz throughout the hysteresis curve, an M61WK pre-amplifier, a data acquisition card, and a PC running the LabVIEW graphical programming software. A spectrum analyzer (SRS780, Stanford Research Systems, Inc., Sunnyvale, CA, USA) with two pre-amplifiers was also included for regression-based spectral analysis. The spectral analysis of the signals yielded a linear regression curve approximating the overall range of the acquired signals. The chemical composition of the specimens is presented in Table 1.

**Figure 2:**
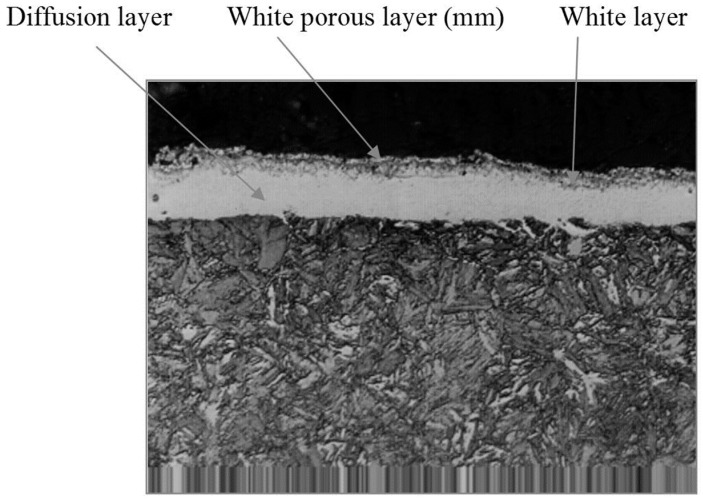
The microstructure of the surface of a 35CrNiMo06 steel sample after nitriding (etched with 2% nital, 500× magnification).

The results of the nitriding treatments were determined by slicing each test sample and then determining the penetration depth of the nitrogen into the surface layer by measuring its Vickers microhardness. The nitriding depth was defined as the depth at which the hardness (in units of HV) was 50% greater than that of the core. Regarding the microstructure (Figure 2), the thicknesses of the white layer and the white porous layer were also measured. The results obtained for 35CrNiM06 steel samples are shown in Table 1 (the microstructures of other materials differ in their specific values but not in their qualitative microstructural characteristics).

**Figure 3:**
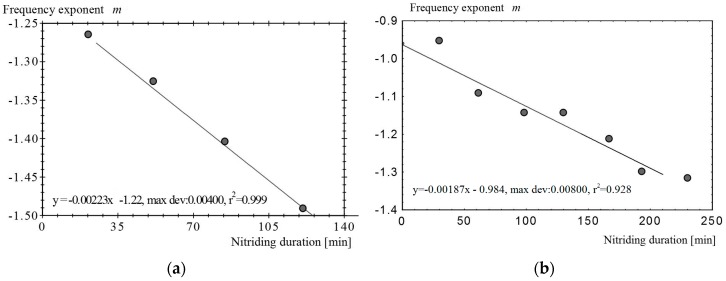

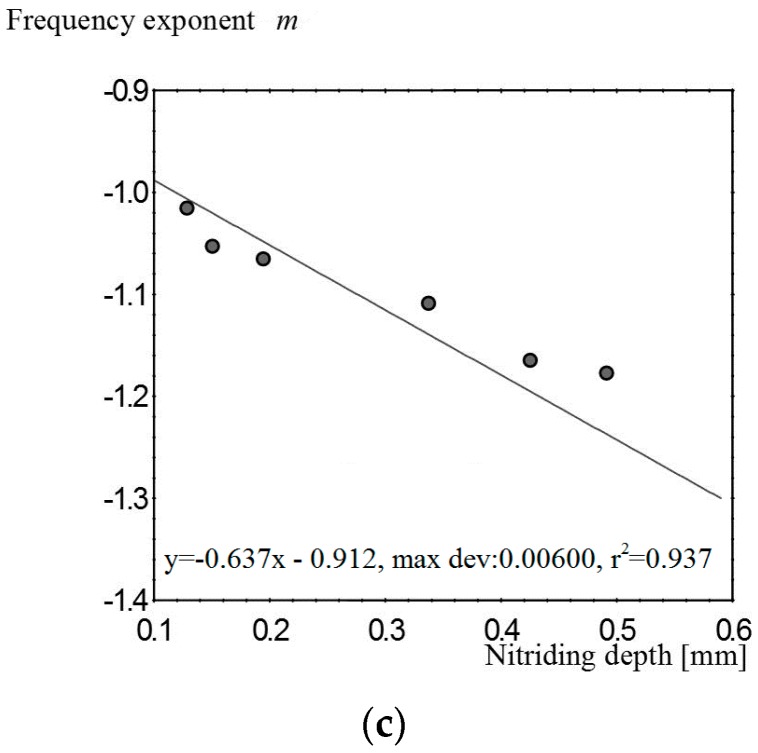
(**a**,**b**) The dependence of the frequency exponent on the treatment duration and the nitriding depth (**a**) when the samples were treated during the same cycle and were successively extracted and (**b**) when the samples were treated in independent cycles with different durations; (**c**) The linear dependence of the frequency exponent on the nitriding depth.

The soaking time during nitriding was treated as the independent variable, whereas the dependent variable was the frequency exponent *m*. The relationship between these two variables was determined in two sets of experiments (Figure 3):

(a) All samples were simultaneously introduced into the furnace, which was programmed for a single nitriding cycle, and the samples were removed after various treatment times;
(b) The samples were individually treated in complete cycles of various durations.

A linear dependence of the frequency exponent on both the nitriding duration and the penetration depth is apparent (Figure 3c).

## 3. Results and Discussion

**Figure 4:**
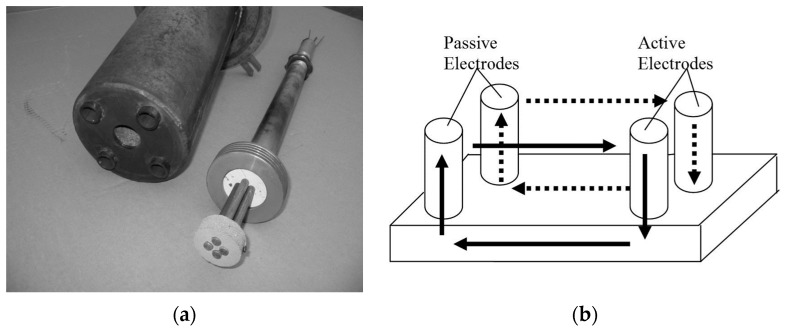

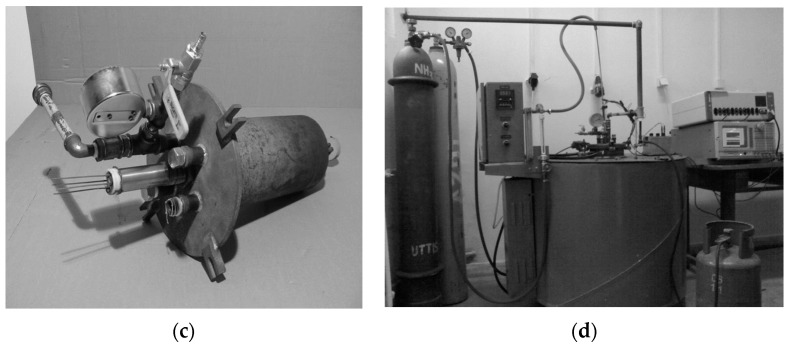
Sensor design: (**a**) experimental construction; (**b**) operating principle; (**c**) sensor in the flange prior to assembly in the furnace; and (**d**) furnace assembly for the experiment.

Based on the preliminary results presented above, the in situ thermal diffusion process was studied. Because of the uncontrollable response of steel to magnetic excitation near the selected nitriding temperature of 580 °C, conductivity noise was used instead of Barkhausen noise. The sensor consisted of two active electrodes (made of steel being subjected to nitriding) and two passive reference electrodes (made of stainless steel that had been nitrided to saturation for 56 h) (Figure 4).

The conductivity noise measured at the passive electrodes was not influenced by the diffusion process, whereas the noise measured at the active electrodes varied as a function of diffusion. The distinctive characteristic of this system is its relation to the properties of the nitriding atmosphere, which consists of atoms and ions and thus conducts electrical signals and creates an electrical circuit (see the arrows in Figure 4b). Because of the nature of this electrical circuit, the electrodes were placed as close to each other as possible using a gauged ceramic disk, which also isolated the electrodes from one another (Figure 4a). This system was positioned (Figure 4c) in the flange of a vertical nitriding furnace (Figure 4d).

**Figure 5:**
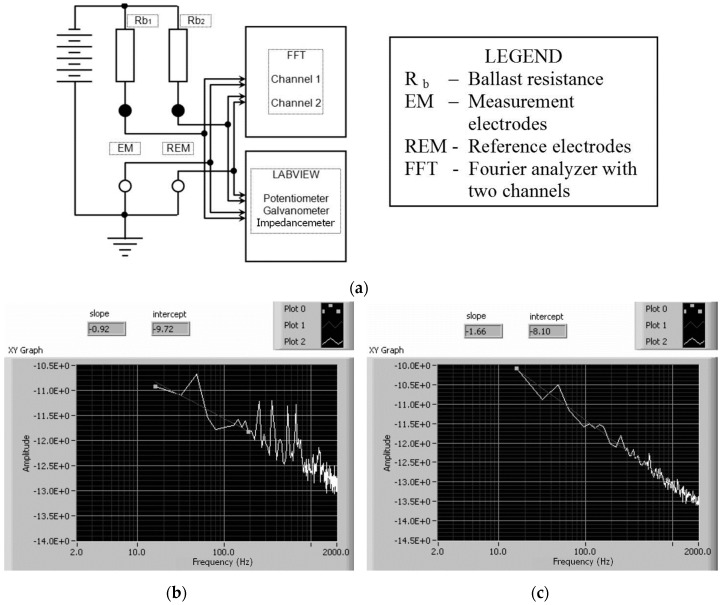
Real-time measurement system: (**a**) the measurement scheme; (**b**) the noise spectrum after one hour of nitriding; and (**c**) the noise spectrum after six hours of nitriding.

The external connections of the system were cooled to a temperature acceptable for connection to the measuring instruments. The complete unit, including the cover, electrodes, polarization devices, equipment, etc., is depicted in Figure 4d. The system, consisting of the electrodes and polarization devices, was connected to a two-channel dynamic signal analyzer (SRS780, Stanford Research Systems, Inc.) and an potentiostat/galvanostat (EcoChemie Autolab PGSTAT30, Utrecht, The Netherlands). Computer control was implemented by means of an application developed in LabVIEW (Figure 5).

The four electrodes could be polarized by a direct current at a voltage between 3 V and 18 V (in steps of 3 V). The electrode polarization system was shielded to reduce the network noise along the signal path. The measurement system (Figure 5) provided a means of signal analysis (FFT analysis) using a Stanford Research SRS780 dynamic analyzer, simultaneously covering the levels of the reference electrodes and those of the electrodes measuring the progress of the nitriding process. The noise spectrum in the range of 16–2000 Hz was recorded at hourly intervals.

To ensure a sufficiently high surface area for ammonia dissociation, the furnace was charged with a ballast composed of pipes with clean surfaces defatted with alcohol. After the chamber (Diameter, *D* = 0.2 m, height, *H* = 0.5 m, Volume, *V* = 0.0157 m^3^) was sealed, it was flushed with a nitrogen flow of 150 L/h for 30 min. Once the oven had reached 300 °C, a flow of ammonia (50 L/h) was supplied. Once a temperature of 520 °C was reached, the dew point had dropped below 20 °C; consequently, the intensity of the reduction reactions began to decrease. After 1.5 h at this temperature, the atmosphere in the furnace contained ammonia and dissociated ammonia (2NH_3_ = N_2_ + 3H_2_). The nitriding potential, which was defined based on the composition and morphology of the desired white layer, was adjusted by increasing or reducing the flow of ammonia. At the end of the process, the treated batch was cooled along with the oven while maintaining the flow of ammonia down to a temperature of 400 °C to produce a shiny metal surface. Before the furnace was opened, it was flushed with nitrogen at a flow rate equal to five times the volume of the oven. For safety reasons, during the process, the oven was maintained at a pressure 0.5–1.5 mbar greater than that of the environment using a super-pressure valve.

Figure 5b,c shows clean spectra of the 1/*f* type, corresponding to frequency index values of 0.92 for 1 h of nitriding (Figure 5b) and 1.66 for 6 h of nitriding (Figure 5c). Table 2 presents the parameters of the noise spectra measured over 8 h of diffusion at intervals of 1 h. Simultaneously, the impedances of the noise-normalization electrodes were measured using the PGSTAT30. The LabVIEW software enabled the readout of the noise spectra and the calculation of the noise parameters (i.e., the noise constant *C*_1/*f*_ and the frequency exponent *m* for various nitriding stages) (Table 2).

**Figure 6:**
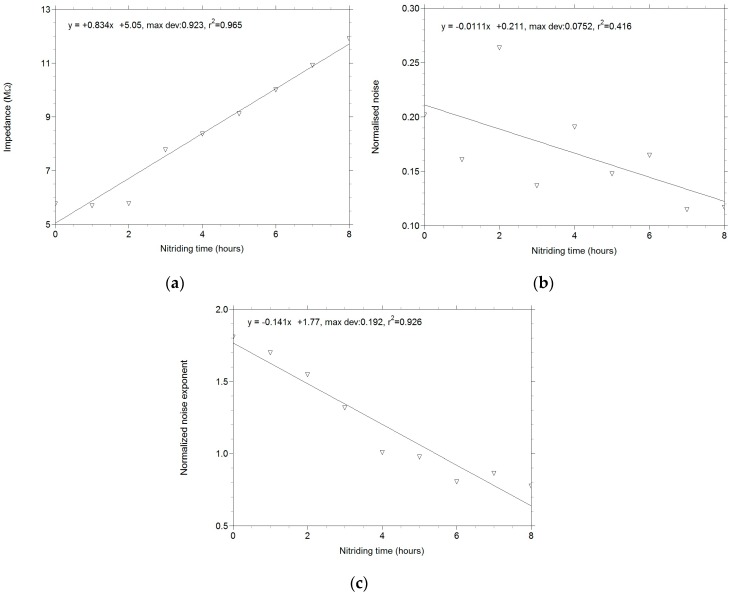
Variations in noise/fluctuations with the nitriding time: (**a**) impedance; (**b**) normalized noise; (**c**) normalized noise exponent.

The graphical presentation of the normalized noise (Figure 6b) shows that early in the diffusion process, when microstructural changes insignificantly affected the measured impedance values, there is a large spread of the measured points around the regression line. As the process continues, microstructural modifications become more important (the nitrogen content in the surface layer increases, changing the microstructure and, consequently, the noise values). The distance between the points and the regression line diminishes, representing a continuous increase in measurement accuracy. The graphical presentation of the variation in the frequency exponent *m* as a function of the nitriding time (Figure 6c) shows the same phenomenon. The linearity of the behavior is maintained, thereby confirming the validity of the experiments based on Barkhausen noise, a finding of utmost importance.

## 4. Conclusions

- An experiment was conceived to investigate the possibility of using electronic noise and fluctuations as a means of tracking in situ diffusion processes in general and the nitriding process in particular.
- A material undergoing a diffusion treatment will exhibit a permanent change in impedance due to changes in the chemical composition of its surface layer (in the specific case considered in this paper, these changes are caused by an increase in the amount of nitrogen in this layer). This change in impedance causes the conduction noise to be proportional to the amount of diffused nitrogen, which was the main motivation for this project.
- The proposed sensor is a circuit consisting of four electrodes. The reference electrodes consist of a material whose chemical composition will not be further modified by diffusion treatment. The other two electrodes, made of the same steel as that of the furnace charge, will exhibit changes in impedance, thereby allowing the conduction noise of the circuit formed by the electrodes (a circuit that is closed by the atmosphere of the furnace) to be recorded and calibrated as a function of the amount of diffused nitrogen.
- Experiments proved that the frequency exponent *m* that was selected as the parameter to be measured to evaluate the evolution of the nitriding process is a reliable tool for this purpose because of its sensitivity to nitrogen-diffusion-induced microstructural changes.
- The linear relationship between the frequency exponent *m* and the diffusion progress offers good accuracy in predicting the quantity of diffused nitrogen, a useful parameter for monitoring the rather sophisticated process of nitriding.
- The extension of the noise analysis from magnetic excitation (Barkhausen noise) to conduction noise has proven to be highly reproducible.
- Future research should address the correlation between the microstructures formed as a result of diffusion and the electrical signals generated by the sensor. We expect that the proposed measurement technology will enable not only the monitoring of the evolution of the nitriding process but also the prediction of the microstructure that will result from the nitriding treatment (compound layer, porosity, etc.).

## Figures and Tables

**Table 1:** Specimen composition and diffusion regime.

Composition of the Steel Used in the Barkhausen Experiments									
Element	%C	%Si	%Mn	%P	%S	%Cr	%Mo	%Al	%Ni
35CrNiM06	0.32–0.39	0.1–0.5	0.5–0.8	Max 0.03	Max 0.035	1.3–1.7	0.15–0.30	-	1.3–1.7
Diffusion regimes used in the experiments									
35CrNiM06 sample (under a protective gas flow of 9 m^3^/h)									
Nitriding time (min)			80			100	160		
Diffusion layer (mm)			0.3100			0.3800	0.4500		
White layer (mm)			0.0130			0.0140	0.0180		
White porous layer (mm)			0.0065			0.0025	0.0080		

**Table 2:** Measured noise and fluctuation parameters.

Nitriding Time (h)	Sensor		Normalized Noise Exponent	Normalized Noise	Impedance MΩ
	Slope	*C*_1/*f*_			
0	−1.17	−6.46	1.81	0.202	5.77
1	−0.92	−9.72	1.70	0.161	5.71
2	−1.53	−9.02	1.55	0.264	5.79
3	−1.07	−10.32	1.32	0.137	7.79
4	−1.60	−8.49	1.01	0.191	8.37
5	−1.36	−8.93	0.978	0.148	9.13
6	−1.66	−8.10	0.808	0.165	10.02
7	−1.26	−9.47	0.866	0.115	10.93
8	−1.40	−9.26	0.777	0.117	11.91
